# Screening-level ecological risk assessment (SLERA) in an abandoned As–Cu mining area (N Spain): implications of phyto-availability and soil properties on vegetation

**DOI:** 10.1007/s10653-025-02394-z

**Published:** 2025-02-22

**Authors:** Humberto Serrano-García, Miguel Izquierdo-Díaz, Fernando Barrio-Parra, Rodrigo Álvarez, Almudena Ordóñez, Eduardo De Miguel

**Affiliations:** 1 Prospecting and Environment Laboratory (PROMEDIAM), E.T.S. de Ingenieros de Minas y Energía, Universidad Politécnica de Madrid, c/Alenza 4, 28003 Madrid, Spain; 2 Departamento de Explotación y Prospección de Minas, Universidad de Oviedo, Gonzalo Gutiérrez Quirós, 33600 Mieres, Spain

**Keywords:** Trace elements, Ecological risk, Abandoned mines, Phyto-availability

## Abstract

This study applies a Screening-Level Ecological Risk Assessment (SLERA) to evaluate the potential impact of trace elements on vegetation in an abandoned As–Cu mining area in northern Spain. A total of 27 soil samples were analyzed for pseudo-total (*aqua regia*) and phyto-available (EDTA 0.05 M) content of As, Cd, Co, Cr, Cu, Ni, Pb and Zn. Contamination and ecotoxicological risk indices were used to identify pollution hotspots and contaminants of ecological concern (COPEC). Arsenic, Cu, Co and Zn pose a potential risk to vegetation across the site. Available concentrations of these elements are high, although average phyto-availabilities are generally low (< 30%). Remarkably, some highly contaminated areas support vegetation, while others remain barren. This suggests that high phyto-available concentrations alone do not fully explain the presence or absence of vegetation. Edaphic properties, such as low soil pH and minimal organic matter content, seem to play a critical role in inhibiting vegetation growth in certain areas. These results underline the importance of considering not only trace element concentrations but also site-specific edaphic properties when assessing ecological risk and planning remediation strategies in contaminated mining areas.

**Supplementary Information:**

The online version contains supplementary material available at 10.1007/s10653-025-02394-z.

## Introduction

According to the United Nations Environment Programme (UNEP, 1999), one of the main environmental problems that affect the mining industry is that of abandoned sites without a restoration management plan. The potential costs of remediation, the lack of clearly assigned (or assumed) responsibilities, the absence of remediation criteria and standards, and other factors have meant that many such sites have been “orphaned” and, until recently, no action had been taken to try to evaluate and mitigate the environmental and health impacts that they potentially entail. Recent reports estimate that there are more than one million abandoned mining areas around the world (Martínez-López et al., 2021; Venkateswarlu et al., 2016) and an undetermined fraction of them may pose an unacceptable risk to ecosystems or nearby populations (Camizuli et al., 2018; Kim et al., 2008).

Once the mining activity ceases, the main source of toxic trace elements is the accumulation of wastes discarded during the extraction of the minerals of interest, since as the separation process is not completely effective the wastes also contain a fraction of ore and at least another ton of waste is generated for every ton of product extracted (Lottermosser, 2010). In addition to trace element enrichment, mining also generates impacts on the environment by altering the natural geomorphology of the area. This leads to landscape impacts and, in general, to a significant increase in erosion processes, especially in steeply sloping tailings. This process can lead to the mobilization and accumulation of non-cohesive material that still contains significant amounts of Potentially Toxic Elements. Water quality can also be affected by past mining operations. Leaching and percolation are one of the main pathways by which abandoned mine sites contaminate local surface and groundwater (Lange et al., 2010). Trace element inputs into aquatic ecosystems can also occur during heavy rainfall events that release large amounts of pollutants into surface and groundwater bodies (Barrio-Parra et al., 2020; Venkateswarlu et al., 2016). This is especially relevant in the current context of climate change, due to the increased recurrence of such extreme weather events (Jentsch & Beierkuhnlein, 2008).

The eventual transfer of these inorganic contaminants to the biosphere is also well documented (Álvarez et al., 2003; Freitas et al., 2004; Moreno-Jiménez et al., 2009). The adverse effects of trace element contamination on ecosystems surrounding mining areas have been extensively studied (Álvarez et al., 2003; Chopin & Alloway, 2007; Kicińska & Wikar, 2021). Screening-Level Ecological Risk Assessment (SLERA) is a useful tool for providing a preliminary estimate of the potential risk due to the transference of these pollutants to ecosystems (USEPA, 1997). SLERA is typically structured in two steps: (i) the screening-level problem formulation and characterization of ecological effects (Step 1) and (ii) the screening-level exposure estimation and risk calculation (Step 2). These steps include the identification of potential receptors, the selection of screening ecotoxicity values, the preliminary identification of contaminants of potential ecological concern (COPECs), and the calculation and characterization of risk.

Plants are specially interesting receptors in the ecological risk assessment because they play a vital role in most terrestrial ecological systems (Markert, 1992). Their ecological importance derives from the fact that they are the basis of terrestrial trophic networks and are consumed by a wide variety of organisms. The potentially negative effect on vegetation of trace elements present in soil is related to the phyto-availability of these elements (Song et al., 2004), so their estimation is a relevant factor when conducting an ecological risk assessment. SLERA is based on ecotoxicological reference values that are often derived from general literature data, and do not reflect the specific conditions at the site under study (US EPA, 1997). This considerably increases the level of uncertainty in risk assessment, as they rarely consider critical factors such as variability in local phyto-availability, the influence of soil properties or the nature of the surrounding plant community, among others. It is undeniable that the natural variability of these properties cannot be adequately accounted for by generic values. Determining the site-specific phyto-availability of trace elements can provide relevant information that is not always considered in traditional risk assessments. In addition, soil properties such as pH and organic matter content significantly influence the availability of heavy metals (Zeng et al., 2011), which adds another dimension to the risk estimation.

Assessing site-specific phyto-availabilities can be approached through two distinct methodologies. On the one hand, it can be determined by analyzing the concentration of the trace element directly in plant tissue. Alternatively, it can be defined as the fraction of the total amount of the element potentially absorbable by plant roots, estimated by a chemical extraction procedure that attempts to simulate the conditions of the rhizosphere environment. The latter methods are frequently preferred because of the reduced complexity and duration of the assays. Since the extractant solutions should have little reactivity with soil components, neutral salts are often used for this purpose (Manzano, 2013). Examples of neutral salts are calcium chloride (CaCl_2_), sodium nitrate (NaNO_3_), ammonium acetate (NH₄CH₃CO₂), and lithium nitrate (LiNO_3_), although the use of other extractants, such as magnesium chloride (MgCl_2_), acetic acid (CH₃COOH) or ethylenediaminetetraacetic acid (EDTA) is also recommended (Abedin et al., 2012).

This study presents an integrated approach that combines pollution and risk indices with detailed phyto-availability and edaphic analyses, offering a novel perspective on the impact of heavy metals on terrestrial ecosystems. To the best of the authors’ knowledge, no attempts have been made to compare Screening-Level Ecological Risk Assessments with site-specific phyto-availability at abandoned mine sites where concentrations of toxic elements are as high as those found at the selected study site. This gap in literature highlights the significance of understanding the relationships between ecological risk and phyto-availability in highly contaminated environments. Consequently, the objectives of this study were to: (i) perform a preliminary estimation of the SLERA for the terrestrial plant community; (ii) assess the applicability scope and limitations of SLERA by considering site-specific factors such as phyto-availability, the influence of certain soil properties (pH and organic matter) and the historical mining and waste management practices; and (iii) explore the role of these parameters on the plant colonizing capacity, thereby contributing to a more comprehensive understanding of the ecological impacts of trace elements in mining-affected ecosystems.

## Materials and methods

### Study area, sampling and preparation

The area of study is located in the province of Palencia (northern Spain), within the so-called Pisuerga-Carrion unit of the Cantabrian Mountains. This region features metasomatic contact (skarn-type) mineralization over carboniferous limestones, influenced by intrusive quartz diorite and tonalite (Martín-Izard et al., 1986). The primary mineral paragenesis includes sulfides and metallic oxides such as arsenopyrite (FeAsS), chalcopyrite (CuFeS_2_), pyrite (FeS_2_, sometimes with pyrrhotite mineral inclusions), magnetite (Fe_3_O_4_) and hematite (Fe_2_O_3_).

The area was primarily exploited for Cu and, to a lesser extent, As through underground operations across five levels of extraction, reaching approximately 100 m in vertical development. Significant mining activity took place during the 1950s, culminating in a peak production of 533 tons of Cu and 209 tons of As in 1952 (Martín-Izard et al., 1986; SIEMCALSA, 2007). The mining complex covers approximately 5000 m^2^ and included its own processing plant, which was essential for extracting As and Cu. This processing involved various separation methods, including washing and roasting. The sedimentation of processing waste, often rich in heavy metals and other contaminants, has contributed to soil and water pollution in the surrounding area, located less than 1 km from a major reservoir with a capacity of 66 Mm^3^, which supplies drinking water to local municipalities.

**Fig. 1:**
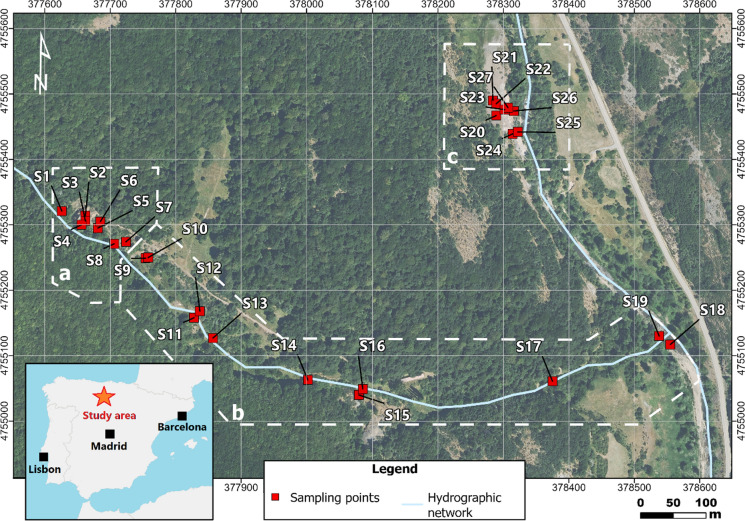
Location of soil samples at the study area divided into three sampling zones: **a** main mining zone; **b** creek zone; **c** processing plant zone. Coord. UTM ETRS89 30N

For the purposes of this work, the study area was divided into three sampling zones (Fig. 1): (a) the main mining area, (b) an area with spoil heaps scattered along a seasonal creek that flows into the main river course of the region, and (c) the ruins of the mineral processing plant, located on the west bank of the river and connected to the mining area by a 700 m long tunnel. Zones (a) and (b) are densely forested, while zone (c) consists of barren anthropogenic backfills (Fig. 1).

A total of 27 soil samples (plus one duplicate) were collected with a stainless-steel hand auger: 8 in the main mining area (zone a), 11 along the creek (zone b) and 8 in the land around the processing plant of zone c (Fig. 1). Samples were transferred to sealed polyethylene bags for transport to the laboratory, where they were oven-dried at 105 °C for 48 h. Dry samples were disaggregated with a rubber mallet and sieved with a 2 mm mesh plastic sieve. Lastly, each sample was subdivided into two subsamples to determine trace element concentrations of the fraction below 100 µm, and edaphic properties (pH and organic matter). This fraction was selected due to its relevance in risk assessments and because finer fractions tend to concentrate higher amounts of trace elements due to their greater specific surface area for ion exchange adsorption (Ljung et al., 2007).

### Chemical analysis

Pseudo-total contents were determined following an *aqua regia* extraction protocol (adapted from ISO 54321:2021): 1.5 g of dry sample was transferred to a polypropylene tube, 1 mL of deionized water and a mixture of 10.5 mL of HCl and 3.5 mL of HNO_3_ were added, and the solution was allowed to stand for 16 h. The solution was then heated to 95 °C for 2 h in a graphite block digestion system, filtered through ALBET No. 240 quantitative paper and made up to 50 mL with 1% HNO_3_.

The phyto-available contents were determined with 0.05 M Na_2_EDTA adjusted to pH 7 with NaOH (following the procedure of Lo & Yang, 1999): 50 mL of this solution were added to 5 g of each soil sample in an Erlenmeyer flask and then placed for one hour in a thermostatized bath at 25 °C with agitation at 100 rpm. The solutions were subsequently centrifuged at 2000 G for 4 min to decant most of the solid particles and, finally, the samples were filtered for further measurement.

All analytical solutions were analyzed by ICP-OES (PerkinElmer Avio 200) to determine the concentrations of 8 trace elements (As, Cd, Co, Cr, Cu, Ni, Pb and Zn). A quality assurance and quality control protocol were implemented to evaluate the accuracy and precision of the extraction and analysis methods: blanks and replicates were analyzed, a certified reference material (ISE sample 995 of Sandy Soil, WEPAL) were extracted and analyzed, and ICP-OES quality controls were also implemented. Each sample was measured 3 times and certified standards were introduced at the beginning, after every 10th sample and at the end of each batch to detect possible cross-contaminations and calibration line drifts. Recovery factors for the soil reference material ranged between 90 and 110% for all elements.

### Pollution indices and hazard quotient

The contamination factor ($CF$) quantifies the degree of soil contamination due to one element as the enrichment relative to its geochemical natural background (Ferreira et al., 2022):

1 $$CF = \frac{{C_{t} }}{{C_{b} }}$$

where ${C}_{t}$ is the total concentration of an element in a soil sample and ${C}_{b}$ is the baseline value. In the absence of local background values, a combination of baseline levels from comparable geological contexts (Adriano, 1986; Loredo et al., 2006) were used instead (Table 1). The natural enrichment of some of the elements, characteristic of this type of mineralization, has been considered. For instance, the background value for As (126 mg kg^−1^) corresponds to average soils overlying sulfide deposits (Adriano, 1986) and data for Cu (24.4 mg kg^−1^) is has been taken from a nearby sulfide ore (Loredo et al., 2006).The $CF$ classifies soil pollution in four categories (Boitshwarelo et al., 2022): $CF$< 1 low contamination; 1 < $CF$< 3 moderate contamination; 3 < $CF$< 6 considerable contamination; $CF$> 6 very high contamination.

**Table 1:** Summary (arithmetic mean x̄, and standard deviation σ) of aqua regia concentrations (mg kg^−1^) of trace elements in soils, and their comparison with geochemical background levels (Adriano, 1986; Loredo et al., 2006; Roberts & Gunn, 2014) and the safe ecological screening values (No Effect ELS) for generic plants (LANL, 2020)

		As	Cd	Co	Cr	Cu	Ni	Pb	Zn
Background		126.0*	0.3*	8.0***	16.6*	24.4**	40*	27.1**	86.2**
No Effect ESL		18.0	32.0	13.0	–	70.0	38.0	120.0	160.0
*Mining area*	x̄	12,186.2	6.8	41.8	19.8	6745.5	32.0	97.8	434.1
	σ	10,274.8	3.6	32.1	4.6	7827.6	14.4	118.3	344.7
*Creek*	x̄	1311.5	3.0	16.5	17.8	1237.6	20.4	14.4	198.9
	σ	1732.0	0.9	3.8	4.0	956.0	8.2	8.5	83.1
*Processing plant*	x̄	34,726.0	8.9	48.0	42.3	5594.8	22.2	21.2	259.4
	σ	11,597.0	4.1	46.0	65.2	3707.4	14.4	12.8	153.7

*Adriano (1986)

**Loredo (2006)

***Roberts and Gunn (2014)

For mixtures of contaminants, one of the most frequently used indices is the pollution load index ($PLI$); (Agyeman et al., 2022; Mandour et al., 2021; Santos et al., 2020). The $PLI$ evaluates the degree of pollution based on the potential contribution of a set of $n$ elements in soil, sediment, or water sample, as:

2 $$PLI = \sqrt[n]{{\mathop \prod \limits_{i = 1}^{n} CF_{j} }}$$

where, $C{F}_{j}$ is the contamination factor for each element $j$. The $PLI$ classifies soil contamination into five levels (Chen et al., 2015): $PLI$ ≤ 1 unpolluted; 1 <$PLI$ ≤ 2 moderately to unpolluted; 2 <$PLI$ ≤ 3 moderately polluted; 3 <$PLI$ ≤ 4 moderately to highly polluted; 4 <$PLI$ ≤ 5 highly polluted; $PLI$> 5 very highly polluted.

The hazard quotient $(HQ)$ expresses the ratio between a potential exposure level (e.g., the pseudo-total concentration in soil) and a toxicological reference value that represents a safe level of exposure (Eq. 3), and it can be used in SLERA to estimate whether the occurrence of harmful effects due to the presence of a pollutant is likely or not (US EPA, 1997):

3 $$HQ = \frac{{C_{t} }}{ESL}$$

where ${C}_{t}$ is the concentration of an element in soil and $ESL$ is an ecological screening level derived from toxicity reference values. For SLERA, US EPA (1997) recommends the No-Adverse Effects Level. In this study, No Effect $ESL$ for generic terrestrial plants were taken from the ECORISK DATABASE (LANL, 2020) (Table 1). The $HQ$ classifies hazard contamination as follows (US EPA, 1997): $HQ$< 1 Harmful effect are not likely; $HQ$= 1 Contaminant alone is not likely to cause ecological risk; $HQ$> 1 Harmful effect cannot be ruled out.

### Phyto-availability and edaphic properties

The mean phyto-availability of trace elements was estimated for the whole site with the following equation (Eq. 1):

4 $$\hat{\beta } = \frac{{\mathop \sum \nolimits_{i = 1}^{n} x_{i} y_{i} \omega_{i} }}{{\mathop \sum \nolimits_{i = 1}^{n} x_{i}^{2} \omega_{i} }}$$

where $\widehat{\beta }$ represents the average phyto-availability (-) of a trace element,${x}_{i}$ and ${y}_{i}$ the *aqua regia* and EDTA concentrations (mg kg^−1^) of sample $i$, respectively and ${\omega }_{i}$ is a weighting factor which can be assumed to be either constant, proportional to ${1/x}_{i}$, or proportional to ${1/x}_{i}$ ^2^ (Izquierdo et al., 2015).

The organic matter content of the soil was determined in the < 2 mm size fraction with potassium dichromate (K_2_Cr_2_O_7_) and subsequent titration with ammonium ferrous sulfate (Mohr’s salt) following the Walkley and Black (1934) method.

The pH was measured on the < 2 mm size fraction according to the standard operating procedure (SOP) of the Division of Research Safety of the University of Illinois (UIUC, 2021): 20 mL of 18.2 MΩ cm^−1^ water were added to 20 g of dry soil (1:1 m/v ratio), the mixture was shaken for 30 min and then it was allowed to stand for 1 min before the pH of the supernatant was measured.

The assessment of the acid mine drainage (AMD) potential was conducted through a visual analysis of mineral abundances under a microscope, where the proportion (in %) of sulfides versus carbonates was estimated.

### Data analysis

The data obtained from the chemical and edaphic analysis of the soils were processed and statistically analyzed using R (R Core Team, 2021), using “stats” library. To assess the dependency relationships between trace elements, Spearman correlation matrices were applied, allowing for the identification of significant correlations between the concentrations of different elements in the soil samples using the *cor()* function. Additionally, to identify patterns of similarity between the soil samples and group them based on their chemical composition, a hierarchical clustering analysis was conducted using the *hclust()* function, employing Euclidean distance as the dissimilarity metric and Ward’s method for clustering.

Variability in trace element concentrations and edaphic properties between the different sampling zones was evaluated through analysis of variance (ANOVA). Subsequently, Tukey’s Honest Significant Differences test was applied for multiple comparisons of individual means, using the *TukeyHSD()* function. This allowed for the identification of statistically significant differences between the mean concentrations of trace elements and their phyto-availability across the different zones of the study site.

For the assessment of the acid mine drainage, ABATES software, supported by the International Network for Acid Prevention (INAP, 2009), was used to calculate the Acidity Producing Potential (APP), Acid Neutralizing Capacity (ANC), and Net Acidity Producing Potential (NAPP) in kg H_2_SO_4_ per ton of material.

## Results and discussion

The complete analytical results of the *aqua regia* extractions can be found in Table A1 (Appendix 1). Table 1 presents a statistical summary of the pseudo-total contents of trace elements in soil (n = 27). Arsenic and Cu were the two most abundant elements in all three zones. The rest of the elements (Cd, Co, Cr, Ni, Pb and Zn) showed mean concentrations between 1 and 4 orders of magnitude lower. A strong correlation between As and Cu has been found (Spearman correlation coefficient ρ = 0.83; Figure A1, Appendix 2), as result of the close association between arsenopyrite and chalcopyrite in this type of mineralization (Dai et al., 2022). Both metallic sulfides are very abundant in this mineralization, and they are closely associated from a textural point of view (Álvarez-Quintana et al., 2020).

Despite the strong correlation between both elements, significant spatial variability in As concentrations was observed across the three zones. A multiple-contrast test (Tukey’s test) revealed significant differences in mean As contents between the mining area, the creek, and the processing plant (p-value < 0.05), while no significant differences were found in mean Cu concentrations (Figure A3, Appendix 2). The As variability can be explained by the historical waste management practices at the site; the As in the mining area proceeds from the arsenopyrite disseminated around the site, whereas in the processing plant, it is released during the roasting of the sulfides, remaining in the immediate soil or being retained in the resulting slags. Despite this variability, the high correlation between the two elements indicates that their common geological source prevails over the observed differences, thus reflecting their strong mineral association (Dai et al., 2022).

The pseudo-total concentrations of Cu and As found in the mining area and processing plant are consistent with the local geological-mining context. During the operation of the mine, unprocessed low-grade ore was dumped around the mine shaft, resulting in a soil Cu content of 0.67% and high concentrations of As (above 1%). The higher presence of As in the soils can be explained by the greater abundance of arsenopyrite in the mining area compared to chalcopyrite. In fact, SEM studies have shown that arsenopyrite crystals are more frequent than Cu species. This arsenopyrite is generally more weathered than chalcopyrite, showing oxidation rims that are depleted in As compared to the cores of the arsenopyrite grains.

In the mining area, the high contents of As and Cu correspond mainly to a mechanical dispersion mechanism. Both elements primarily exist in the soils in the form of primary minerals, which may somewhat mitigate the associated risk. However, as one moves downstream from the mining area, mechanical dispersion quickly loses intensity, with only small particles of sulfides present; at this point, dispersion is dominated by chemical mechanisms. In terms of associated risk, this area shows a higher proportion of As and Cu adsorbed onto mineral surfaces (silicates or carbonates, and probably organic matter), which are more bioavailable forms than the primary ones.

In this regard, the creek could play an important role as a transport medium for As and Cu to the impounded waters of the reservoir. Although it is beyond the scope of this work to assess the Cu and As contents in water, it seems that the creek’s contribution to the reservoir is not very significant. The concentrations in the soil decrease by up to an order of magnitude in just a few tens of meters. This decrease can be attributed, in part, to dilution effects caused by runoff, which help reduce contaminant concentrations in the soil. Similar downstream dilution effects in soils and sediments of riverine environments have been documented by other authors (Cabrera et al., 2008; Grosbois et al., 2001).

Around the processing plant, the average As concentration in the soil is a remarkable 3.5%, as a consequence of a beneficiation process in which As was not fully recovered and remained partly in the waste material of the plant. Even after the beneficiation of Cu, the direct disposal of the residues from the processing plant has resulted in slightly lower concentrations in the soil compared to the mining area (0.56%). Both concentrations are respectively 3 and 2 orders of magnitude higher than the corresponding ecological safety values for generic plants. This situation is common at such sites, as the old extractive metallurgical operations were clearly improvable, and very little attention was generally paid to environmental aspects. Since Cu was the object of production, the process was designed to optimize its recovery, which has resulted in a relatively low intensity of Cu-derived pollution. However, the As released during the roasting of the sulfides remains in the immediate soil or is retained in the resulting slags. These slags, which have been sampled and examined, contain enormous proportions of As, but their vitreous texture suggests that they should be inert in terms of reactivity.

**Fig. 2:**
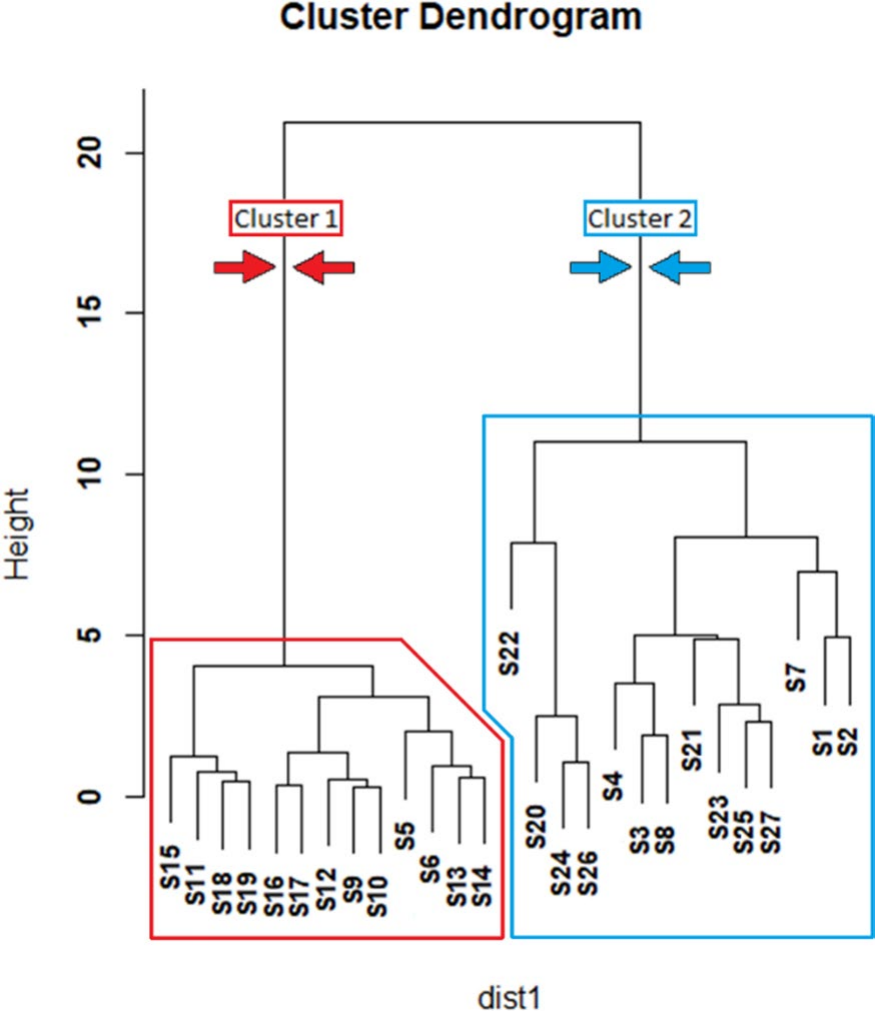
Cluster Analysis (linkage method: Ward, distance: Euclidean) of soil and sediment samples (cluster 1 in red, and cluster 2 in blue)

A cluster analysis of the samples performed with the *hclust()* function of the “stats” library (R Core Team, 2021) revealed that soil samples could be classified in two main groups (Fig. 2). The first cluster is composed mainly of samples collected on both margins of the creek, affected by naturalized spoil heaps scattered along the creek and to downstream areas unaffected or only lightly affected by mining operations. The second cluster is made up of most of the samples near the main mining area and all the samples from the processing plant. The association between these two zones is consistent with the findings of the multiple contrast test for Cu and is consistent with the fact that the main mining area was directly connected to the processing plant through a 700 m long excavated ditch through which the material was transported for processing (Martín-Izard et al., 1986).

### Ecological risk assessment

**Fig. 3:**
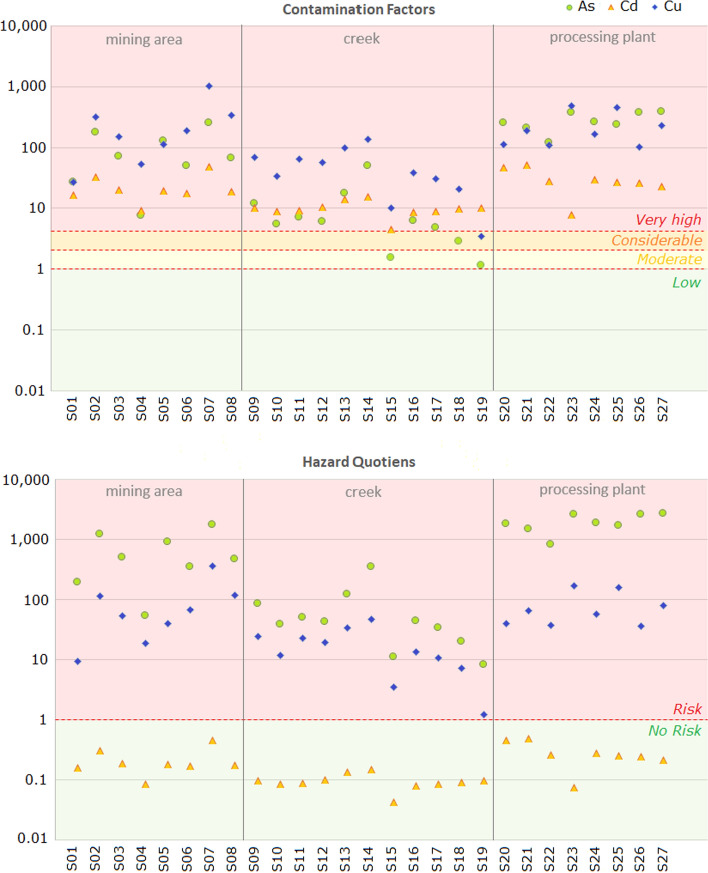
Comparative view between contamination factors (above) and hazard quotients (below) for As, Cd and Cu

As a first step in SLERA, the contaminants of most concern among those present at the site were identified (USEPA, 1997; Efroymson et al., 1997). To evaluate the contamination levels of the eight trace elements, the mean contamination factors ($CF$) were calculated. The results showed that Cu had the highest $CF$ (170.5), followed by As with a $CF$ of 114.5. Cadmium also presented a high $CF$ value of 19.6, while Co, Zn, Cr, Pb and Ni exhibited lower $CF$ values: 4.2, 3.3, 1.5, 1.5 and 0.6, respectively. According to the $CF$ scale (Boitshwarelo et al., 2022), the analyzed soils were very highly contaminated with Cu, As and Cd ($CF$> 6), considerably contaminated with Co and Zn (3 < $CF$< 6), and moderately contaminated with Cr and Pb (1 < $CF$< 3). Figure 3 provides information on the degree of contamination associated with As, Cd and Cu and its variability. Although Cu and As show a strong variability (coefficients of variation CV = 113 and 125%, respectively), they are found in most of the samples in the range of very high contamination (82% for As and 96% for Cu). Cadmium was less heterogeneous (CV = 66%), but in most cases (96%) it was within the same range. Except for specific cases, the rest of the elements (Co, Cr, Ni, Pb and Zn) remained at low to moderate contamination levels (Table A2, Appendix 1).

**Table 2:** Comparison of average phyto-availability (%) for the whole site using three different mathematical models

	As	Cd	Co	Cu	Ni	Pb	Zn
Model 1 $\omega_{i} constant$	0.9***	20.9**	8.6***	12.4***	10***	6.8***	8.3***
Model 2 $\omega_{i} \propto 1/x_{i}$	0.9***	23.6**	12.7***	13.5***	11.8***	15.3***	8.6***
Model 3 $\omega_{i} \propto 1/x_{i}^{2}$	1.2***	26.9**	19.7***	15.7***	13.8***	29.2***	9.0***

p-value***< 0.001*; and *< 0.01

**Fig. 4:**
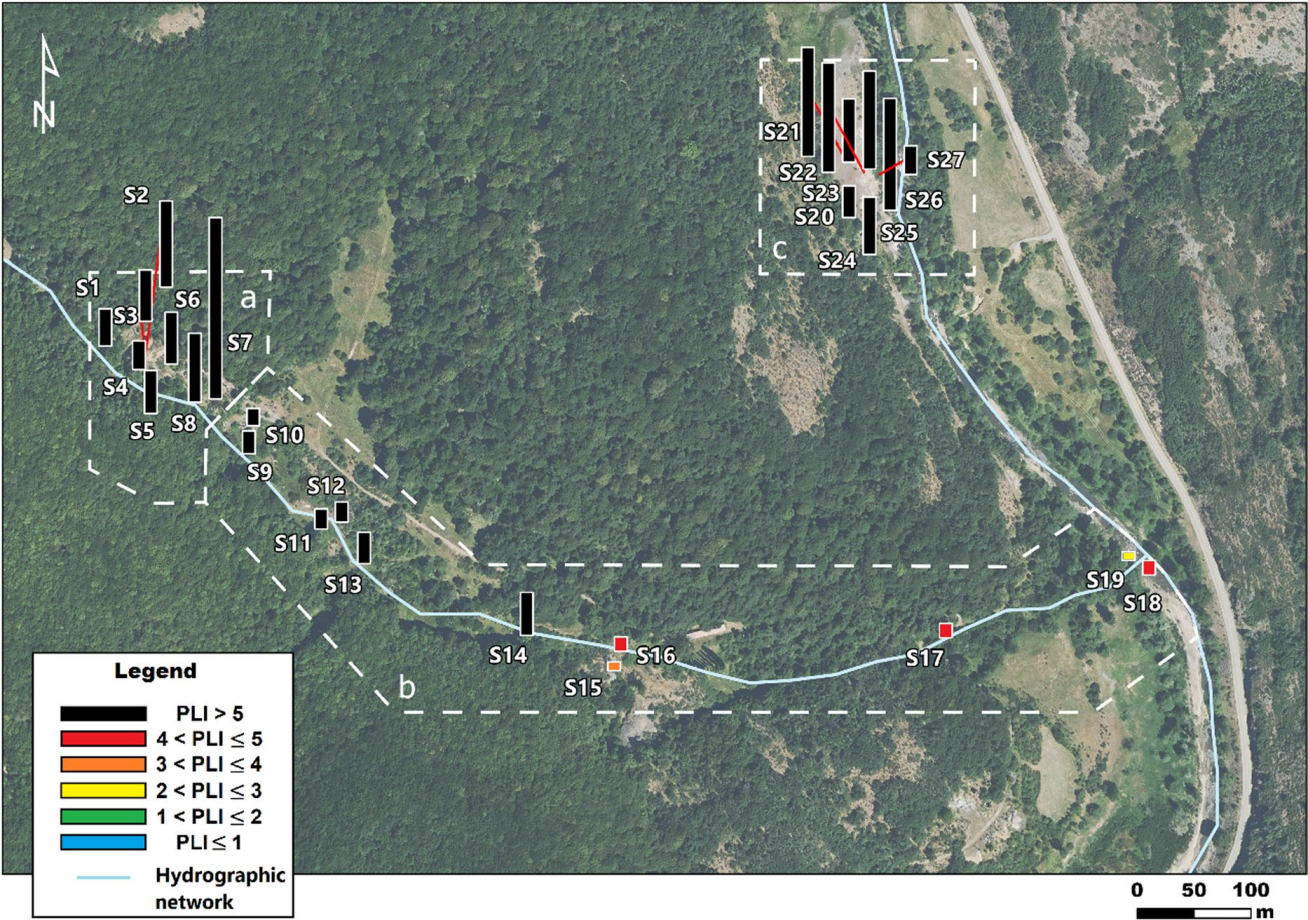
Spatial distribution of the soil pollution load index (PLI). PLI ≤ 1 unpolluted soil; 1 < PLI ≤ 2 moderately to unpolluted soil; 2 < PLI ≤ 3 moderately polluted soil; 3 < PLI ≤ 4 moderately to highly polluted soil; 4 < PLI ≤ 5 highly polluted soil; PLI > 5 very highly polluted soil (Chen et al., 2015). **a** main mining zone; **b** creek zone; **c** processing plant zone

Following Tomlinson (1980), the $PLI$ was calculated considering a maximum of five elements (in this case, those with the highest mean $CF$: As, Cd, Co, Cu and Zn). The results indicated very high levels of contamination ($PLI$> 5) throughout the site, especially in the main mining and processing plant zones (Fig. 4). Moderate to high levels ($PLI$ between 3 and 5) were only detected in the eastern sector of the creek, close to the mouth (S15 to S19). None of the samples could be classified as unpolluted soil.

The $PLI$ can be a useful tool to identify hot spots of soil contamination. This concept usually refers to a specific area where contaminant concentrations are significantly higher than in the surroundings (Hadley & Mueller, 2012). However, it has been challenging to establish specific hot spots at this site, given that the entire area is heavily degraded. According to criteria established by the Interstate Technology & Regulatory Council (ITRC, 2008), hot spots can be defined as areas where mean contaminant concentrations are between 10 and 100 times higher than those in the surrounding area. In this context, the concentrations of certain elements, such as As and Cu, are sufficient to classify the mining area and the processing plant zones as hot spots (Table 1). Nevertheless, a qualitative analysis of the spatial distribution of the $PLI$ leads to the same conclusion (Fig. 4).

Despite the generalized low soil geochemical quality, the creek lower course and mouth present lower levels of contamination than the headwater area (Fig. 4). This suggests that, although the creek soils are strongly influenced by contamination from the mining area, this impact diminishes downstream. As mentioned above, this decrease can be attributed, in part, to dilution effects caused by the runoff. The contamination gradient observed along the creek may positively impact the overall ecological health by enhancing the colonization capacity of vegetation, which is consistent with the notable development of vegetation observed in this area (Fig. 1).

$CF$ and $PLI$ do not allow to infer the risk of exposure of an organism or an ecosystem to a set of pollutants. For this purpose, it is necessary to use other indicators that include ecotoxicological information. Following US EPA (1997) recommendations for SLERA, $HQ$ for each pollutant were calculated at each sampling point (Table A2, Appendix 1). As this study focuses on the ecological risk for plants, phyto-toxicological reference levels (No Effect ELS) provided by the ECORISK DATABASE (LANL, 2020) were used. The mean $HQ$ for the eight trace elements were: As (801.8) > Cu (59.4) > Co (2.6) > Zn (1.8) > Ni (0.6) = Cr (0.6) > Pb (0.3) > Cd (0.2). Values greater than 1 are indicative of potential adverse effects to the receptor whereas values equal to or less than 1 indicate that the risk from exposure to that element is negligible and that it can therefore be disregarded as a COPEC.

These results suggest that, from a phyto-toxicological point of view, As and Cu are the elements that can cause the most damaging effects in plants, followed by Co and Zn. Cd can be excluded from the list of COPECs at the site, despite its high degree of contamination in soil (Fig. 3). It is well known that Cu is one of the metals that most affects plant growth (Mondaca et al., 2017). Although, it is an essential element for higher plants (especially important for photosynthesis), its excess in soils can induce stunted plant growth and other harmful effects (Nagajyoti et al., 2010). Arsenic has no known biological function in plants and, although some taxa have developed mechanisms to retain most of the As in their root systems, its presence can affect their morphology, physiology and metabolic processes (Bali & Sidhu, 2021; Finnegan & Chen, 2012). Cobalt is an essential element for some plant groups, such as legumes (Mahey et al., 2020), but levels in soils above 100 mg kg^−1^ can already have observable phyto-toxic effects (Nessim & Abdalla, 2008). Finally, Zn can also easily accumulate in plants and lead to severe growth disturbances or cause metabolic disorders (Bazihizina et al., 2014; Kaur & Garg, 2021).

Of particular concern is the proximity of the abandoned mine and, especially, of the processing plant to the nearby 66 Mm^3^ reservoir, which serves as a source of drinking water for several municipalities in the region. This proximity raises significant concerns regarding the potential transfer of soil contaminants to the adjacent river sediments and water column where they would not only pose a threat to aquatic ecosystems but would also have implications for human health. Given the importance of the reservoir as a source of drinking water, future studies focusing on river sediments and their interaction with the water column will provide a more complete understanding of the environmental risks associated with this region.

### Phyto-availability, soil pH and organic matter content

To explore how local environmental conditions may influence the degree of exposure of receptors, phyto-availability has also been estimated and its dependence on site-specific parameters (soil pH and organic matter) has been evaluated. The complete analytical results of the EDTA extracts are presented in Table A3 (Appendix 1), together with their corresponding phyto-availability values (i.e. the ratio of EDTA extractable contents and the pseudo-total content). To provide a first approach to the degree of availability of trace elements to plants, their mean phyto-availabilities have been estimated for the whole site. They are summarised in Table 2, which also shows the p-values of β for the 3 models (considering β = 0 as the null hypothesis). These estimators were significant for all elements. No significant differences were observed between the three models (the estimation of the mean phyto-availability is not conditioned by the method used), except for Co and Pb. The differences in these two elements are a consequence of the greater influence of low values in the estimates with models 2 and 3, whereas in model 1 all data exert equal weight (Izquierdo et al., 2015). The mean phyto-availabilities (Table 2) follow the order: Cd (20.9–26.9%) > Cu (12.4–15.7%) > Ni (10.0–13.8%) > Zn (8.3–9%) > As (0.9–1.2%). The position of Co (8.6–19.7%) and Pb (6.8–29.2%) in this list depends on the calculation method used. The phyto-availability of Cr could not be evaluated since all the EDTA extracts presented concentrations of this element below the detection limit. The strong retention of Cr in the crystalline structure of the mineral fraction (Angelone et al., 1990) and the difficulty to form the Cr(III)-EDTA complex at room temperature (25 °C) (Baraud & Leleyter, 2012; Yotova et al., 2018) may explain this circumstance.

**Fig. 5:**
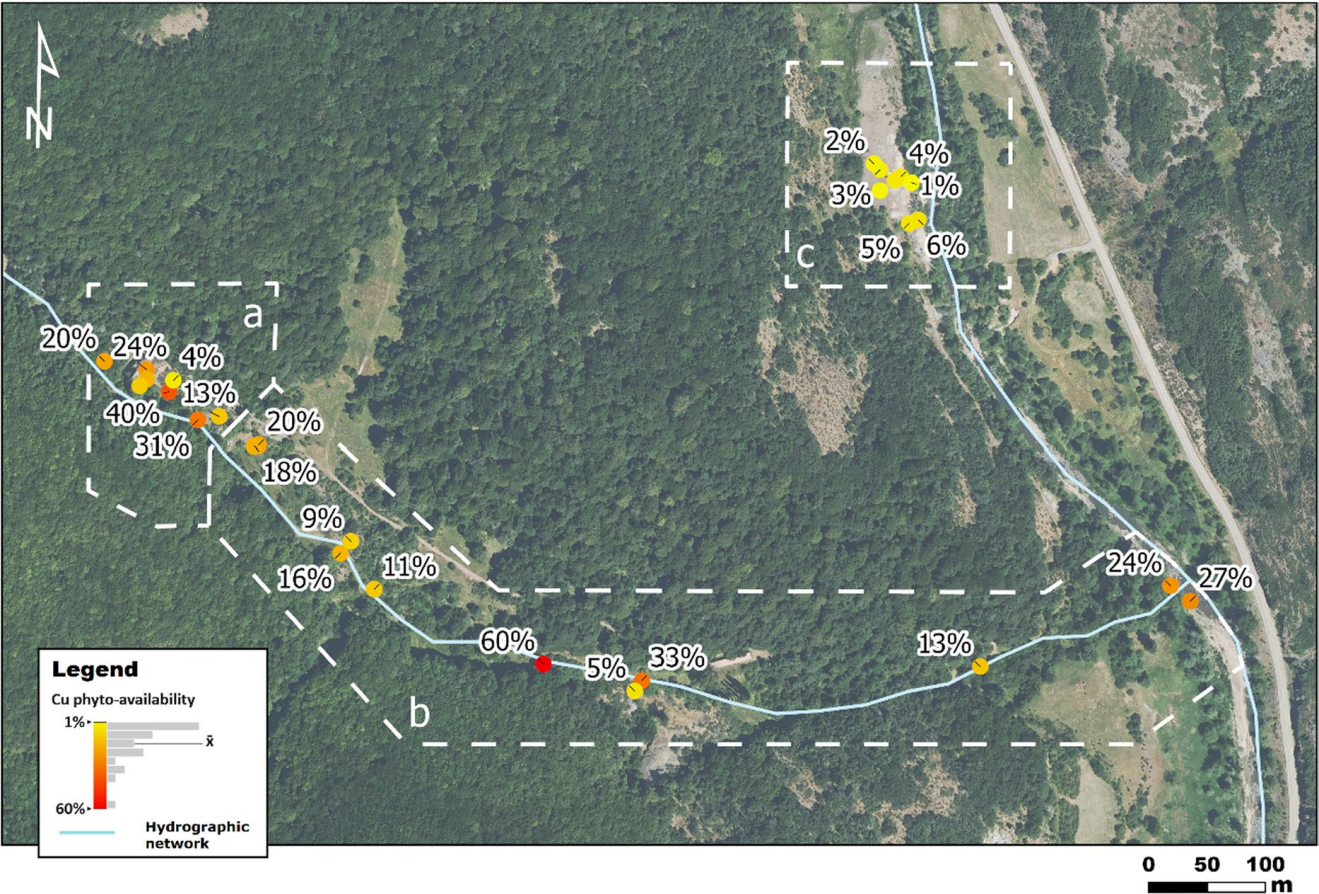
Spatial distribution of Cu phyto-availabilities (%). **a** main mining zone; **b** creek zone; **c** processing plant zone

Mean values are a first approach to understand the site-specific phyto-availability but, on their own, they do not capture the strong variability that characterizes this parameter in the study area (Table A3, Appendix 1). For instance, Cd and Cu were the most phyto-available elements, but they showed a large variability (CV = 77% and 88%, respectively). As noted above, risk assessment identified Cu as a COPEC for plants. According to Berrow and Reaves (1985), EDTA contents higher than 30 mg kg^−1^ could induce plant growth deficiencies. The average phyto-available Cu concentration in the soils analyzed far exceeds this threshold (563 mg kg^−1^). However, a spatial analysis of the data revealed that Cu phyto-availability is not homogeneously distributed (Fig. 5), with the lowest values (maximum 6%) found in the soils of the mineral processing plant. In comparison, in the mining and creek areas they are, in general, one order of magnitude higher (maximum 60%). A Tukey test (p-value < 0.05) revealed significant differences between the processing plant and the other two zones (Figure A4, Appendix 2). Although some authors have suggested a direct relationship between the amount of Cu in the soil and its potential lability (Kabata-Pendias & Pendias, 2001; Yotova et al., 2018), this behavior has not been observed in the samples analyzed. A plausible explanation for this divergence could lie in the textural differences between the soils in these two zones. As mentioned above, soils from the mineral processing plant contain mainly small fragments of slag. These materials tend to have vitreous textures that may hinder Cu mobilization. Wastes from the mining and creek zones, however, consist of host rock fragments, frequently with some sulfide mineralization unstable under oxidizing conditions, which would release Cu more easily.

Organic matter content and pH of soils are the most important parameters affecting the availability of heavy metals (Zeng et al., 2011). To assess the influence of these parameters on Cu availability, a Spearman analysis was performed (Figure A2, Appendix 2). Due to the limited data on Cu availability in the processing plant subpopulation (n = 7), it was considered to combine these data with those from the mining area (n = 15 in total) prior to correlation analysis (Figure A2, Appendix 2). The results indicated that there is not a significant correlation between pH content and Cu phyto-availability (ρ = 0.15), but there is regarding organic matter (ρ = 0.69). A significant decrease in Cu availability has been observed when the organic matter content is also reduced. The analysis revealed that the soils with the lowest organic matter values (processing plant, mean 0.1%) correspond to the lowest phyto-availabilities (mean 3%). Opposite, in soils with higher organic matter contents (main mine and creek, mean 6%), significantly higher phyto-availabilities were obtained (mean 21%). A possible explanation could be found in the high affinity of Cu for organic matter (Bolan et al., 2003) compared to other less soluble soil fractions. Therefore, in soils at the study site with low organic matter, Cu is bound to less available fractions, such as (oxy) hydroxides and primary minerals, for which the extraction capacity of EDTA is much more limited. Since EDTA extraction releases a large amount of metals retained in the organic fraction, the phyto-availability of this element in soils with low organic content can be expected to decrease significantly.

Cobalt and Zn showed very similar mean phyto-availabilities (Table 2). The behavior of both elements is like that described for Cu. Significant differences were observed between the processing plant and the other two zones (p-value < 0.05), and positive correlations were established with organic matter content (ρ = 0.60 and 0.67, respectively). However, correlations with pH turned out to be rather weak (ρ = 0.20 and 0.01, respectively).

Arsenic was the least phyto-available element (Table 2). Although some variability was also observed, a Tukey test (p-value < 0.05) revealed that there are no significant differences between the three zones (Figure A4, Appendix 2). The slight differences observed could be due to several factors. Neither pH nor organic matter content seems to play an important role in this case (ρ = −0.29 and 0.31). This is consistent with the observations made by Wenzel (2013), who suggested that as extracted by EDTA does not come mainly from the organic phases of the soil. Its low phyto-availability also could be explained by the difficulty to form stable complexes with EDTA (Přibil, 1982). Is known that the mineralogical composition of the soil can influence the availability of the metalloid (Meunier et al., 2010). For instance, the presence of as from arsenopyrite represents one of its least available forms (< 1%). Conversely, its availability increases when it is part of alteration minerals as Fe-(oxy) hydroxides or Fe-arsenates (1–10%). The results of this study indicate that most of the as comes from arsenopyrite. It is therefore to be expected that the lowest phyto-availabilities are found in areas that have undergone less alteration, whereas in areas more exposed to weathering, as availability increases.

A final aspect to consider is how the influence of all these parameters translates into the observable adverse effects on the plant community. Although some areas considered at risk for exposure to COPECs ($HQ$ > 1) are sterile (the processing plant), others show abundant vegetation (the main mine and the creek). It is known that stress caused by soil acidity can have a negative effect on plant growth (Zhao et al., 2014). This observation could provide an initial guide to understanding variations in forest density at the site.

Samples with the lowest pH values are concentrated in the barren area of the processing plant (S20, S24, S26 and S27; Table A3, Appendix 1). According to the U.S. Department of Agriculture (USDA, 2017) classification, these soils can be categorized as ultra-acidic and extremely acidic (pH < 3.5 and 3.5–4.4, respectively). In contrast, the mining area and the creek, which are densely forested, have an average pH around 6 (moderately acidic soils) and even reach a slight degree of alkalinity at some points (pH > 7). However, samples S2, S5 (mining area) and S12 (creek) show a high degree of acidity (Table A3, Appendix 1), despite being in areas that are generally forested. It is relevant to note that these three samples were collected in spoil heaps with no vegetation.

A Tukey test revealed that differences in pH values between the processing plant and the other two zones are significant at a 90% confidence level (Figure A5, Appendix 2). The lower acidity observed in the mine and creek appears to be the result of the neutralizing effect of the higher carbonate content in the mineral fraction of these soils compared to the slags. The mineralization, formed through contact metamorphism on a calcareous olistolith (Martín-Izard et al., 1986), contributes to this phenomenon, allowing the pH in these areas to be higher compared to that of the processing plant.

The overall conditions in the area are predominantly acidic and may be explained by acid mine drainage, especially in the zones of accumulation of mining waste, where the rocks are approximately 35% mineralized, primarily consisting of 33% arsenopyrite, 33% chalcopyrite, 20% pyrite, and 14% other sulfides. On the other hand, the host rock (65%) consists of 75% silicates and 25% calcite. Analysis using ABATES software indicates that this mineral composition leads to an Acidity Producing Potential (APP) 3.6 times greater than the Acid Neutralizing Capacity (ANC), resulting in a Net Acidity Producing Potential (NAPP) of 316 kg of H_2_SO_4_ per ton of material. A sample with NAPP > 0 must be classified as potentially acid generating (Smith et al., 1992). Consequently, acid generation is likely to pose a significant issue for rocks containing this mineralogy.

Although the current observations do not yield definitive conclusions, they suggest that the highly acidic conditions prevailing in much of the processing plant (possibly in combination with the absence of organic matter) may be limiting effective plant colonization in this specific area of the site. However, it is important to note that these initial observations require further analysis and consideration of other factors (e.g., a floristic inventory, detailed mineralogy of soil mineral fraction…) to fully understand the relationship between soil pH and the presence of vegetation in the study area.

## Conclusions

A Screening-Level Ecological Risk Assessment (SLERA) has been performed to evaluate the potential impact of trace elements on the plant community in a former As–Cu mining complex. The use of pollution indices, in combination with eco-toxicological risk indices, has allowed to identify the areas of highest trace element accumulation and the COPECs. Soil in the area occupied by the main mining operation and the former mineral processing plant are of most concern ($PLI$> 5) and can be considered as hot spots. Lower contaminant loads in the eastern sector of the creek suggest that the metal content in soil is rapidly diluted downstream before reaching the river. On the other hand, the $HQ$ estimations suggest that concentrations of As, Cu, Co and Zn pose a potential risk to vegetation on the whole site ($HQ$> 1) and should be considered as the COPECs.

The scope of the application of ecotoxicological reference values in SLERA has also been evaluated, especially when local conditions diverge from generic values. Although the average phyto-availabilities were low, a large variability was observed along the site. In some cases, this variability seems to correspond to the higher or lower presence of organic matter in the soil. Soil pH had no clear relationship with the phyto-availability of the elements evaluated. The adverse effects observed on the plant community have been contrasted with the results obtained. While high exposure to COPECs alone does not explain the differences observed between deforested and vegetated areas, the importance of soil acidity stress on plant growth has been raised. A relationship between soil acidity (possibly in combination with the absence of organic matter) and the absence of vegetation has been observed. However, these initial observations need further analysis and consideration of other factors to fully understand the relationship between soil pH and the presence of vegetation in the study area.

## Declaration of generative AI and AI-assisted technologies in the writing process

During the preparation of this work, the authors used ChatGPT 3.5 only to improve the readability and language of selected parts of the text. After using this tool, the authors reviewed and edited the content as needed and took full responsibility for the content of the publication.

## Supplementary Information

Below is the link to the electronic supplementary material.

Supplementary file1 (DOCX 44 KB)

Supplementary file2 (DOCX 177 KB)

## Data Availability

Detailed R code employed in this work for statistical analysis is available upon request from H. Serrano-García (https://portalcientifico.upm.es/es/).
