# Supplementary material for: Screening-level ecological risk assessment (SLERA) in an abandoned As–Cu mining area (N Spain): implications of phyto-availability and soil properties on vegetation
Source: Environ Geochem Health. 2025 Feb 22;47(3):85. doi: 10.1007/s10653-025-02394-z (PMC11846780; doi:10.1007/s10653-025-02394-z)
Supplement: Supplementary file 1 — Supplementary file1 (DOCX 44 KB) [file 10653_2025_2394_MOESM1_ESM.docx]

Table A1 Analytical results of aqua regia concentrations of trace elements in soils and sediments of the site.

|  |  | **Coord. 30T** | | **As** | **Cd** | **Co** | **Cr** | **Cu** | **Ni** | **Pb** | **Zn** |
| --- | --- | --- | --- | --- | --- | --- | --- | --- | --- | --- | --- |
| **Zone** | **Sample** | X | Y | *mg kg^-1^* | | | | | | | |
| *Mining area* | S1 | 377626 | 4755321 | 3434.1 | 5.0 | 23.9 | 25.2 | 660.5 | 42.2 | 159.5 | 1042.0 |
|  | S2 | 377662 | 4755314 | 21903.1 | 9.7 | 27.0 | 19.4 | 7836.1 | 52.2 | 360.4 | 360.3 |
|  | S3 | 377662 | 4755306 | 8913.8 | 5.9 | 38.2 | 21.7 | 3706.4 | 38.6 | 117.4 | 183.5 |
|  | S4 | 377656 | 4755300 | 947.7 | 2.7 | 113.2 | 11.7 | 1311.2 | 41.0 | 7.2 | 177.2 |
|  | S5 | 377681 | 4755295 | 16327.0 | 5.8 | 12.5 | 24.1 | 2764.6 | 7.6 | 43.1 | 157.7 |
|  | S6 | 377685 | 4755305 | 6185.9 | 5.3 | 22.9 | 18.2 | 4649.4 | 21.9 | 26.3 | 302.9 |
|  | S7 | 377724 | 4755274 | 31272.2 | 14.3 | 60.0 | 15.3 | 24904.8 | 21.3 | 48.0 | 909.2 |
|  | S8 | 377706 | 4755271 | 8505.8 | 5.6 | 36.4 | 22.5 | 8130.9 | 31.3 | 20.5 | 340.3 |
| *Creek* | S9 | 377754 | 4755249 | 1505.9 | 3.1 | 19.6 | 15.8 | 1701.9 | 18.3 | 7.6 | 149.5 |
|  | S10 | 377758 | 4755250 | 688.0 | 2.7 | 17.2 | 20.3 | 812.9 | 18.2 | 5.6 | 123.4 |
|  | S11 | 377828 | 4755158 | 884.6 | 2.8 | 17.0 | 14.4 | 1582.3 | 26.8 | 6.7 | 160.9 |
|  | S12 | 377837 | 4755168 | 765.7 | 3.1 | 12.7 | 23.9 | 1366.2 | 20.5 | 22.3 | 192.8 |
|  | S13 | 377857 | 4755127 | 2191.0 | 4.2 | 20.6 | 16.7 | 2367.4 | 16.3 | 15.5 | 280.2 |
|  | S14 | 378002 | 4755066 | 6224.0 | 4.7 | 23.4 | 16.1 | 3273.6 | 11.5 | 26.3 | 218.9 |
|  | S15 | 378080 | 4755040 | 196.4 | 1.3 | 12.5 | 25.6 | 244.0 | 26.7 | 29.7 | 375.6 |
|  | S16 | 378083 | 4755046 | 795.7 | 2.6 | 12.0 | 16.8 | 923.0 | 11.7 | 9.7 | 128.2 |
|  | S17A | 378375 | 4755060 | 602.3 | 2.7 | 12.0 | 11.8 | 749.6 | 7.4 | 4.8 | 88.0 |
|  | S17B | 378374 | 4755060 | 732.7 | 2.6 | 12.4 | 12.6 | 754.6 | 11.2 | 6.7 | 87.7 |
|  | S17_x̄_ |  |  | 667.5 | 2.6 | 12.2 | 12.2 | 752.1 | 9.3 | 5.8 | 87.8 |
|  | S18 | 378553 | 4755116 | 363.0 | 2.9 | 16.2 | 16.3 | 505.6 | 30.1 | 13.6 | 259.7 |
|  | S19 | 378538 | 4755130 | 144.9 | 3.0 | 17.8 | 17.4 | 85.0 | 34.6 | 15.9 | 210.8 |
| *Processing plant* | S20 | 378289 | 4755467 | 32288.5 | 14.2 | 0.5 | 17.1 | 2745.7 | 4.9 | 6.4 | 137.5 |
|  | S21 | 378284 | 4755490 | 26536.0 | 15.4 | 129.8 | 15.0 | 4599.6 | 23.4 | 11.9 | 217.0 |
|  | S22 | 378295 | 4755492 | 14937.5 | 8.2 | 28.6 | 203.1 | 2620.1 | 20.8 | 7.9 | 359.4 |
|  | S23 | 378302 | 4755470 | 46738.3 | 2.3 | 62.7 | 32.5 | 11744.4 | 41.2 | 23.3 | 593.0 |
|  | S24 | 378314 | 4755439 | 33172.6 | 8.8 | 11.1 | 15.2 | 4008.5 | 8.4 | 19.7 | 149.9 |
|  | S25 | 378322 | 4755442 | 29948.0 | 8.1 | 83.6 | 19.5 | 10926.1 | 44.2 | 24.1 | 253.3 |
|  | S26 | 378311 | 4755470 | 46262.8 | 7.8 | 0.5 | 16.0 | 2513.0 | 11.6 | 43.9 | 140.2 |
|  | S27 | 378306 | 4755491 | 47924.2 | 6.8 | 66.8 | 19.8 | 5601.0 | 23.2 | 32.4 | 224.6 |

Table A3 Analytical results of EDTA extracts, phyto-available fractions (PA), soil pH and soil organic matter content (OM) (<LOD = below limit of detection).

|  |  | **As** | **Cd** | **Co** | **Cr** | **Cu** | **Ni** | **Pb** | **Zn** | | **As_PA_** | | **Cd_PA_** | **Co_PA_** | **Cu_PA_** | **Ni_PA_** | **Pb_PA_** | **Zn_PA_** | **pH** | | **OM** |
| --- | --- | --- | --- | --- | --- | --- | --- | --- | --- | --- | --- | --- | --- | --- | --- | --- | --- | --- | --- | --- | --- |
| **Zone** | **Sample** | *mg kg^-1^* | | | | | | | |  | | *%* | | | | | | | | *%* | |
| *Mining*  *area* | S1 | 19.9 | <LOD | 3.6 | <LOD | 134.7 | 3.8 | 40.2 | 64.7 | | 0.6 | | - | 15.0 | 20.4 | 9.0 | 25.2 | 6.2 | 6.1 | 3.9 | |
|  | S2 | 45.2 | 0.8 | 1.5 | <LOD | 1859.2 | 2.3 | 6.0 | 43.0 | | 0.2 | | 8.2 | 5.5 | 23.7 | 4.5 | 1.7 | 11.9 | 5.0 | 3.8 | |
|  | S3 | 34.7 | <LOD | 4.2 | <LOD | 622.9 | 1.2 | 11.1 | 13.6 | | 0.4 | | - | 11.1 | 16.8 | 3.2 | 9.5 | 7.4 | 6.3 | 2.1 | |
|  | S4 | 3.1 | <LOD | 15.1 | <LOD | 142.8 | 0.8 | 1.1 | 5.9 | | 0.3 | | - | 13.4 | 10.9 | 2.0 | 15.5 | 3.3 | 6.0 | <0.1 | |
|  | S5 | 125.1 | 2.7 | 6.0 | <LOD | 1094.4 | 2.0 | 7.4 | 27.1 | | 0.8 | | 46.8 | 48.1 | 39.6 | 26.2 | 17.2 | 17.2 | 3.7 | 0.9 | |
|  | S6 | 34.3 | <LOD | 0.8 | <LOD | 181.2 | <LOD | 0.7 | <LOD | | 0.6 | | - | 3.3 | 3.9 | - | 2.6 | 0.0 | 6.1 | 9.6 | |
|  | S7 | 182.8 | 6.8 | 0.4 | <LOD | 3118.3 | <LOD | 11.2 | 97.9 | | 0.6 | | 47.9 | 0.6 | 12.5 | - | 23.2 | 10.8 | 7.1 | 4.7 | |
|  | S8 | 131.5 | 3.1 | 4.8 | <LOD | 2528.6 | 4.3 | 7.0 | 55.3 | | 1.5 | | 55.3 | 13.3 | 31.1 | 13.8 | 33.9 | 16.3 | 6.6 | 3.9 | |
| *Creek* | S9 | 39.7 | 0.0 | 7.9 | <LOD | 303.7 | 5.5 | 3.5 | 26.0 | | 2.6 | | - | 40.4 | 17.8 | 30.0 | 45.6 | 17.4 | 6.1 | 11.7 | |
|  | S10 | 7.0 | <LOD | 5.2 | <LOD | 161.9 | 6.1 | 2.6 | 13.9 | | 1.0 | | - | 30.4 | 19.9 | 33.3 | 47.3 | 11.3 | 5.3 | 10.0 | |
|  | S11 | 13.5 | <LOD | 5.8 | <LOD | 256.6 | 6.1 | 1.6 | 21.4 | | 1.5 | | - | 34.0 | 16.2 | 22.7 | 23.1 | 13.3 | 5.2 | 9.4 | |
|  | S12 | 2.5 | <LOD | 1.1 | <LOD | 128.0 | 2.4 | 3.1 | 13.1 | | 0.3 | | - | 8.8 | 9.4 | 11.5 | 13.9 | 6.8 | 5.1 | 4.1 | |
|  | S13 | 15.7 | <LOD | 3.6 | <LOD | 270.9 | 1.8 | 5.7 | 17.9 | | 0.7 | | - | 17.2 | 11.4 | 11.1 | 37.0 | 6.4 | 7.3 | 2.6 | |
|  | S14 | 341.7 | 8.9 | 6.3 | <LOD | 1980.3 | 1.3 | 8.3 | 24.0 | | 5.5 | | 191.1 | 26.8 | 60.5 | 11.6 | 31.5 | 11.0 | 4.8 | 10.7 | |
|  | S15 | 4.1 | <LOD | <LOD | <LOD | 13.4 | 0.3 | 13.0 | 20.9 | | 2.1 | | - | - | 5.5 | 1.2 | 43.8 | 5.6 | 8.4 | <0.1 | |
|  | S16 | 20.1 | <LOD | 3.7 | <LOD | 300.4 | 2.5 | 4.4 | 21.4 | | 2.5 | | - | 30.7 | 32.5 | 21.3 | 45.0 | 16.7 | 4.4 | 13.8 | |
|  | S17 | 8.4 | <LOD | 4.0 | <LOD | 99.8 | 0.3 | 3.1 | 5.9 | | 1.3 | | - | 32.8 | 13.3 | 3.3 | 53.5 | 6.7 | 5.3 | 0.6 | |
|  | S18 | 12.8 | <LOD | 5.7 | <LOD | 134.5 | 9.5 | 9.1 | 31.5 | | 3.5 | | - | 35.0 | 26.6 | 31.8 | 67.1 | 12.1 | 4.6 | 10.0 | |
|  | S19 | <LOD | <LOD | 6.2 | <LOD | 20.6 | 11.2 | 8.3 | 26.1 | | - | | - | 35.1 | 24.3 | 32.2 | 52.2 | 12.4 | 5.0 | 11.3 | |
| *Processing*  *plant* | S20 | 91.6 | 0.8 | <LOD | <LOD | 32.3 | <LOD | <LOD | <LOD | | 0.3 | | 5.9 | - | 1.2 | - | - | - | 3.2 | <0.1 | |
|  | S21 | 71.5 | 0.4 | 1.7 | <LOD | 102.1 | <LOD | <LOD | 4.2 | | 0.3 | | 2.9 | 1.3 | 2.2 | - | - | 1.9 | 7.1 | <0.1 | |
|  | S22 | 205.6 | 3.6 | <LOD | <LOD | 84.4 | <LOD | <LOD | 15.7 | | 1.4 | | 44.2 | - | 3.2 | - | - | 4.4 | 7.5 | <0.1 | |
|  | S23 | 1583.2 | <LOD | 1.8 | <LOD | 514.4 | 0.2 | 0.7 | 42.0 | | 3.4 | | - | 2.9 | 4.4 | 0.6 | 3.0 | 7.1 | 7.6 | <0.1 | |
|  | S24 | 89.8 | 0.9 | <LOD | <LOD | 212.1 | <LOD | <LOD | 1.1 | | 0.3 | | 10.1 | - | 5.3 | - | - | 0.7 | 3.6 | 0.4 | |
|  | S25 | 66.9 | 0.5 | 9.0 | <LOD | 658.3 | 2.3 | <LOD | 14.3 | | 0.2 | | 6.2 | 10.8 | 6.0 | 5.2 |  | 5.7 | - | <0.1 | |
|  | S26 | 147.1 | 2.1 | <LOD | <LOD | 26.1 | <LOD | <LOD | <LOD | | 0.3 | | 27.0 | - | 1.0 | - | - | - | 3.2 | 0.2 | |
|  | S27 | 169.0 | 2.8 | 8.5 | <LOD | 234.9 | 0.4 | <LOD | 10.9 | | 0.4 | | 41.8 | 12.8 | 4.2 | 1.9 | - | 4.9 | 4.1 | <0.1 | |

Table A2 Contamination factors (CF), pollution load indices (PLI) and hazard quotients (HQ) for trace elements in soils.

|  |  | **As_CF_** | **Cd_CF_** | **Co_CF_** | **Cr_CF_** | **Cu_CF_** | **Ni_CF_** | **Pb_CF_** | **Zn_CF_** | **PLI** | **As_HQ_** | **Cd_HQ_** | **Co_HQ_** | **Cr_HQ_** | **Cu_HQ_** | **Ni_HQ_** | **Pb_HQ_** | **Zn_HQ_** |
| --- | --- | --- | --- | --- | --- | --- | --- | --- | --- | --- | --- | --- | --- | --- | --- | --- | --- | --- |
| **Zone** | **Sample** |  |  |  |  |  |  |  |  |  |  |  |  |  |  |  |  |  |
| *Mining area* | S1 | 27.3 | 16.7 | 3.0 | 1.5 | 27.1 | 1.1 | 5.9 | 12.1 | 13 | 190.8 | 0.2 | 1.8 | 0.6 | 9.4 | 1.1 | 1.3 | 6.5 |
|  | S2 | 173.8 | 32.2 | 3.4 | 1.2 | 321.2 | 1.3 | 13.3 | 4.2 | 30 | 1216.8 | 0.3 | 2.1 | 0.5 | 111.9 | 1.4 | 3.0 | 2.3 |
|  | S3 | 70.7 | 19.8 | 4.8 | 1.3 | 151.9 | 1.0 | 4.3 | 2.1 | 18 | 495.2 | 0.2 | 2.9 | 0.5 | 52.9 | 1.0 | 1.0 | 1.1 |
|  | S4 | 7.5 | 9.1 | 14.1 | 0.7 | 53.7 | 1.0 | 0.3 | 2.1 | 10 | 52.7 | 0.1 | 8.7 | 0.3 | 18.7 | 1.1 | 0.1 | 1.1 |
|  | S5 | 129.6 | 19.3 | 1.6 | 1.5 | 113.3 | 0.2 | 1.6 | 1.8 | 15 | 907.1 | 0.2 | 1.0 | 0.6 | 39.5 | 0.2 | 0.4 | 1.0 |
|  | S6 | 49.1 | 17.6 | 2.9 | 1.1 | 190.5 | 0.5 | 1.0 | 3.5 | 18 | 343.7 | 0.2 | 1.8 | 0.4 | 66.4 | 0.6 | 0.2 | 1.9 |
|  | S7 | 248.2 | 47.7 | 7.5 | 0.9 | 1020.7 | 0.5 | 1.8 | 10.5 | 63 | 1737.3 | 0.4 | 4.6 | 0.4 | 355.8 | 0.6 | 0.4 | 5.7 |
|  | S8 | 67.5 | 18.8 | 4.5 | 1.4 | 333.2 | 0.8 | 0.8 | 3.9 | 24 | 472.5 | 0.2 | 2.8 | 0.5 | 116.2 | 0.8 | 0.2 | 2.1 |
| *Creek* | S9 | 12.0 | 10.3 | 2.5 | 1.0 | 69.8 | 0.5 | 0.3 | 1.7 | 8 | 83.7 | 0.1 | 1.5 | 0.4 | 24.3 | 0.5 | 0.1 | 0.9 |
|  | S10 | 5.5 | 9.0 | 2.2 | 1.2 | 33.3 | 0.5 | 0.2 | 1.4 | 6 | 38.2 | 0.1 | 1.3 | 0.5 | 11.6 | 0.5 | 0.0 | 0.8 |
|  | S11 | 7.0 | 9.3 | 2.1 | 0.9 | 64.8 | 0.7 | 0.2 | 1.9 | 7 | 49.1 | 0.1 | 1.3 | 0.3 | 22.6 | 0.7 | 0.1 | 1.0 |
|  | S12 | 6.1 | 10.4 | 1.6 | 1.4 | 56.0 | 0.5 | 0.8 | 2.2 | 7 | 42.5 | 0.1 | 1.0 | 0.6 | 19.5 | 0.5 | 0.2 | 1.2 |
|  | S13 | 17.4 | 14.0 | 2.6 | 1.0 | 97.0 | 0.4 | 0.6 | 3.3 | 11 | 121.7 | 0.1 | 1.6 | 0.4 | 33.8 | 0.4 | 0.1 | 1.8 |
|  | S14 | 49.4 | 15.6 | 2.9 | 1.0 | 134.2 | 0.3 | 1.0 | 2.5 | 15 | 345.8 | 0.1 | 1.8 | 0.4 | 46.8 | 0.3 | 0.2 | 1.4 |
|  | S15 | 1.6 | 4.5 | 1.6 | 1.5 | 10.0 | 0.7 | 1.1 | 4.4 | 3 | 10.9 | 0.0 | 1.0 | 0.6 | 3.5 | 0.7 | 0.2 | 2.3 |
|  | S16 | 6.3 | 8.5 | 1.5 | 1.0 | 37.8 | 0.3 | 0.4 | 1.5 | 5 | 44.2 | 0.1 | 0.9 | 0.4 | 13.2 | 0.3 | 0.1 | 0.8 |
|  | S17 | 4.8 | 8.9 | 1.5 | 0.7 | 30.7 | 0.2 | 0.2 | 1.0 | 5 | 33.5 | 0.1 | 0.9 | 0.3 | 10.7 | 0.2 | 0.0 | 0.5 |
|  | S18 | 2.9 | 9.7 | 2.0 | 1.0 | 20.7 | 0.8 | 0.5 | 3.0 | 5 | 20.2 | 0.1 | 1.2 | 0.4 | 7.2 | 0.8 | 0.1 | 1.6 |
|  | S19 | 1.2 | 10.2 | 2.2 | 1.0 | 3.5 | 0.9 | 0.6 | 2.4 | 3 | 8.1 | 0.1 | 1.4 | 0.4 | 1.2 | 0.9 | 0.1 | 1.3 |
| *Processing plant* | S20 | 256.3 | 47.4 | 0.1 | 1.0 | 112.5 | 0.1 | 0.2 | 1.6 | 11 | 1793.8 | 0.4 | 0.0 | 0.4 | 39.2 | 0.1 | 0.1 | 0.9 |
|  | S21 | 210.6 | 51.3 | 16.2 | 0.9 | 188.5 | 0.6 | 0.4 | 2.5 | 38 | 1474.2 | 0.5 | 10.0 | 0.3 | 65.7 | 0.6 | 0.1 | 1.4 |
|  | S22 | 118.6 | 27.3 | 3.6 | 12.2 | 107.4 | 0.5 | 0.3 | 4.2 | 22 | 829.9 | 0.3 | 2.2 | 4.7 | 37.4 | 0.5 | 0.1 | 2.2 |
|  | S23 | 370.9 | 7.8 | 7.8 | 2.0 | 481.3 | 1.1 | 0.9 | 6.9 | 38 | 2596.6 | 0.1 | 4.8 | 0.8 | 167.8 | 1.1 | 0.2 | 3.7 |
|  | S24 | 263.3 | 29.4 | 1.4 | 0.9 | 164.3 | 0.2 | 0.7 | 1.7 | 20 | 1842.9 | 0.3 | 0.9 | 0.4 | 57.3 | 0.2 | 0.2 | 0.9 |
|  | S25 | 237.7 | 26.8 | 10.5 | 1.2 | 447.8 | 1.1 | 0.9 | 2.9 | 39 | 1663.8 | 0.3 | 6.4 | 0.5 | 156.1 | 1.2 | 0.2 | 1.6 |
|  | S26 | 367.2 | 26.0 | 0.1 | 1.0 | 103.0 | 0.3 | 1.6 | 1.6 | 10 | 2570.2 | 0.2 | 0.0 | 0.4 | 35.9 | 0.3 | 0.4 | 0.9 |
|  | S27 | 380.4 | 22.6 | 8.3 | 1.2 | 229.5 | 0.6 | 1.2 | 2.6 | 34 | 2662.5 | 0.2 | 5.1 | 0.5 | 80.0 | 0.6 | 0.3 | 1.4 |

s
