# Supplementary material for: Screening-level ecological risk assessment (SLERA) in an abandoned As–Cu mining area (N Spain): implications of phyto-availability and soil properties on vegetation
Source: Environ Geochem Health. 2025 Feb 22;47(3):85. doi: 10.1007/s10653-025-02394-z (PMC11846780; doi:10.1007/s10653-025-02394-z)
Supplement: Supplementary file 2 — Supplementary file2 (DOCX 177 KB) [file 10653_2025_2394_MOESM2_ESM.docx]

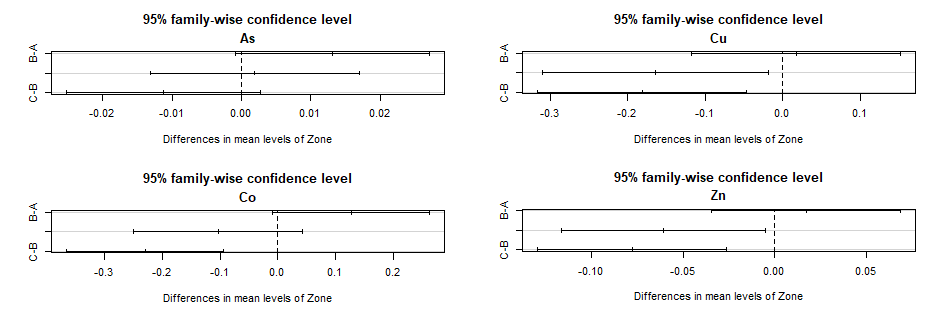

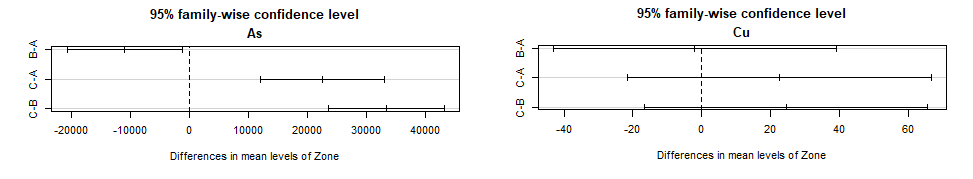


Figure A1 Spearman correlation matrix of pseudo-total trace element contents.


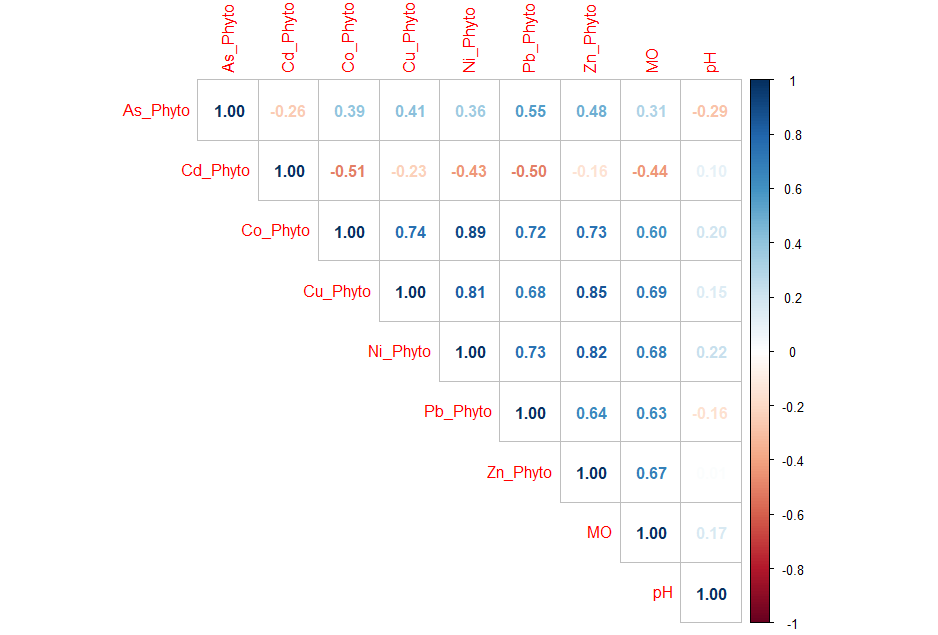


Figure A2 Spearman correlation matrix of trace elements phyto-availabilities, organic matter (MO) and pH.


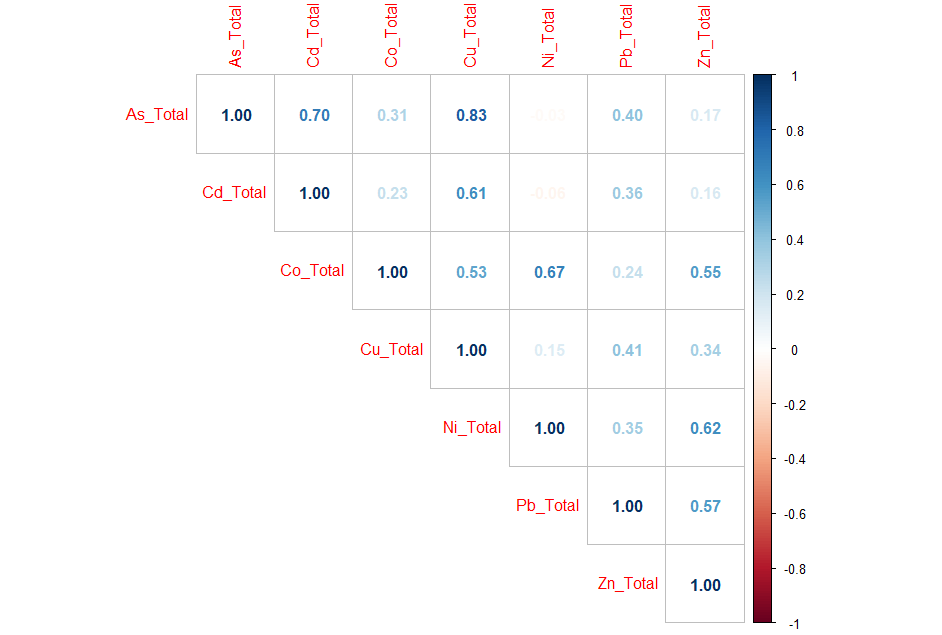


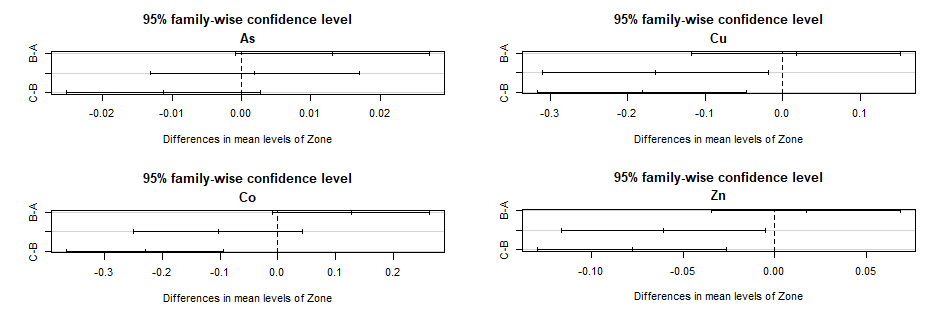
Figure A3 Results of Tukey tests (Honest Significant Differences) for As and Cu pseudo-total content in the three zones (A: mining area, B: creek area, C: processing plant area) at a 95% confidence level (p < 0.05).

Figure A4 Results of Tukey tests (Honest Significant Differences) for the COPEC’ phyto-availabilities in the three zones (A: mining area, B: creek area, C: processing plant area) at a 95% confidence level (p < 0.05)


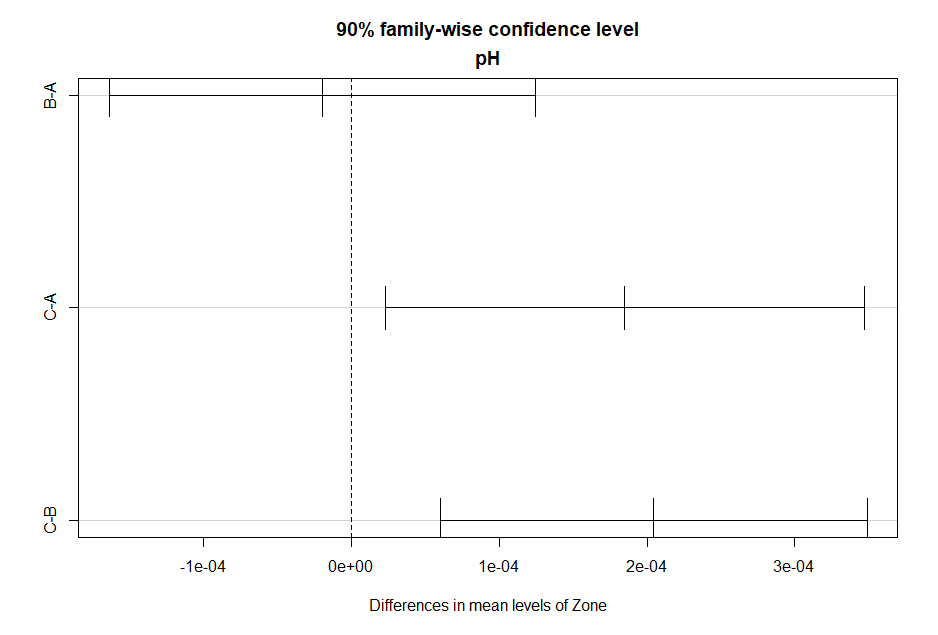


Figure A5 Result of Tukey test (Honest Significant Differences) for the pH of the soil in the three zones (A: mining area, B: creek area, C: processing plant area) at a 90% confidence level (p < 0.1).

s
